# Connectometry reveals differing associations of cortisol and PACAP with dorsal cingulum microstructure in posttraumatic stress

**DOI:** 10.1101/2025.06.05.25329085

**Published:** 2025-06-06

**Authors:** Steven J. Granger, Sydney A. Jobson, Caitlin Ravichandran, Quentin Devignes, Eylül Akman, Jennifer U. Blackford, Victor May, Sayamwong E. Hammack, William A. Carlezon, Kerry J. Ressler, Scott L. Rauch, Isabelle M. Rosso

## Abstract

Posttraumatic stress disorder (PTSD) has been associated with altered arousal regulation and dysfunction of the hypothalamic-pituitary-adrenal axis, including changes in circulating cortisol and pituitary adenylate cyclase-activating polypeptide (PACAP). Both stress-related hormones affect extended amygdala to medial prefrontal cortex (mPFC) circuit functioning, but it is unclear whether they relate to white matter microstructure connecting these regions. We examined this question in 139 trauma-exposed adults (81 female; ages 19-54) who completed the Clinician-Administered PTSD Scale, a blood draw, and diffusion magnetic resonance imaging. White matter integrity was assessed in tracts connecting the extended amygdala to mPFC, including the uncinate fasciculus, frontal parahippocampal cingulum, and bed nucleus of the stria terminalis to mPFC projections. We used both tract-average fractional anisotropy (FA) to assess the global integrity of these white matter tracts and restricted connectometry to identify spatially localized associations along specific tract segments. Neither cortisol nor PACAP levels were associated with tract-average FA in any tract. However, connectometry, using a stringent statistical T-threshold revealed distinct, region-specific associations within the dorsal cingulum: higher cortisol levels were associated with lower FA ( *FDR* =.002), whereas higher PACAP levels were associated with higher FA ( *FDR* =.01). These localized FA alterations were not significantly associated with symptom severity. These findings suggest that cortisol and PACAP levels have differing associations with microstructural integrity of the dorsal cingulum, a region implicated in emotional regulation. These results highlight how distinct stress hormone pathways may differentially impact white matter organization in PTSD and demonstrate the utility of connectometry for detecting regionally specific brain-biomarker relationships.

## Full Text Availability

The license terms selected by the author(s) for this preprint version do not permit archiving in PMC. The full text is available from the preprint server.

